# The Mitochondrial Targets of Neuroprotective Drug Vinpocetine on Primary Neuron Cultures, Brain Capillary Endothelial Cells, Synaptosomes, and Brain Mitochondria

**DOI:** 10.1007/s11064-019-02871-9

**Published:** 2019-09-18

**Authors:** Gergely Svab, Judit Doczi, Akos A. Gerencser, Attila Ambrus, Ferenc Gallyas, Balazs Sümegi, László Tretter

**Affiliations:** 1 Department of Medical Biochemistry, MTA-SE Laboratory for Neurobiochemistry, Semmelweis University, 37-47 Tuzolto Street, Budapest, 1094 Hungary; 2 Buck Institute for Research on Aging, Novato, CA USA; 3 Department of Biochemistry and Medical Chemistry, University of Pecs Medical School, Pecs, Hungary; 4 Szentagothai Research Centre, University of Pecs, Pecs, Hungary; 5 Nuclear-Mitochondrial Interactions Research Group, Hungarian Academy of Sciences, Budapest, Hungary

**Keywords:** Vinpocetine, Neuroprotection, Mitochondria, Reactive oxygen species, Calcium induced calcium release, Oxygen consumption, ATP synthesis, Uncoupling

## Abstract

Vinpocetine is considered as neuroprotectant drug and used for treatment of brain ischemia and cognitive deficiencies for decades. A number of enzymes, channels and receptors can bind vinpocetine, however the mechanisms of many effects’ are still not clear. The present study investigated the effects of vinpocetine from the mitochondrial bioenergetic aspects. In primary brain capillary endothelial cells the purinergic receptor-stimulated mitochondrial Ca^2+^ uptake and efflux were studied. Vinpocetine exerted a partial inhibition on the mitochondrial calcium efflux. In rodent brain synaptosomes vinpocetine (30 μM) inhibited respiration in uncoupler stimulated synaptosomes and decreased H_2_O_2_ release from the nerve terminals in resting and in complex I inhibited conditions, respectively. In isolated rat brain mitochondria using either complex I or complex II substrates leak respiration was stimulated, but ADP-induced respiration was inhibited by vinpocetine. The stimulation of oxidation was associated with a small extent of membrane depolarization. Mitochondrial H_2_O_2_ production was inhibited by vinpocetine under all conditions investigated. The most pronounced effects were detected with the complex II substrate succinate. Vinpocetine also mitigated both Ca^2+^-induced mitochondrial Ca^2+^-release and Ca^2+^-induced mitochondrial swelling. It lowered the rate of mitochondrial ATP synthesis, while increasing ATPase activity. These results indicate more than a single mitochondrial target of this vinca alkaloid. The relevance of the affected mitochondrial mechanisms in the anti ischemic effect of vinpocetine is discussed.

## Introduction

Vinpocetine has been marketed for more than 30 years. Among its indication are ischemic neuronal damage [1], stroke [2, 3], cerebrovascular diseases [4], neurodegenerative diseases [5, 6], dementia and cognitive deficits [1, 7]. During this period many targets and mechanisms of actions has been proposed, including the inhibition of the cyclic nucleotide phosphodiesterase 1 (PDE1) [8, 9], voltage-dependent Na^+^ channels [10, 11], the IκB kinase, the NF-κB [12] and binding to peripheral benzodiazepine receptors [13]. For a recent review see [14]. As a consequence of having numerous targets, the drug can influence diverse functions. The present study focuses on the effects of vinpocetine on mitochondrial function. The brain is extremely sensitive to proper provision of energy, in which mitochodria play a central role. Most of the inborn errors of metabolism are associated with structural and/or functional brain damage and mental retardation. Mitochondria are not only cellular power stations, but also pollute their environment with reactive oxygen species (ROS). Although as a consequence of powerful antioxidant systems most of the mitochondrially produced ROS will not leave the mitochondria in physiological conditions [15], excessive mitochondrial ROS generation is considered as an important factor in cerebral ischemia/reperfusion injury [16–19], neurodegeneration [20] and glutamate toxicity [21, 22]. Early studies already addressed mitochondria as potential vinpocetine targets, but only in a very few studies were mitochondria the focus of the investigation. Vinpocetine’s effect has been explained by binding to the peripheral benzodiazepine receptor [22], a mitochondrial outer membrane protein and putative component of the mitochondrial permeability transition pore (PTP). In the present study the effects of vinpocetine are investigated at three level of complexity, in cellular systems (primary neuronal and endothelial cell cultures), in isolated nerve terminals (synaptosomes) and in isolated guinea pig brain mitochondria. Complex biological phenomena like delayed calcium deregulation, intracellular calcium transients and Ca^2+^ release from in situ mitochondria have been investigated in cellular systems, while mitochondrial oxygen consumption, ROS production, ATPase activity in isolated mitochondria. We conclude that the delayed PTP opening, mild mitochondrial depolarization and decreased H_2_O_2_ release may all play an important role in the beneficial effects of vinpocetine in pathological conditions associated with neuronal damage.

## Materials and Methods

### Animals

For cell culture and synaptosomal experiments Wistar rats were used. Mitochondria were isolated from guinea pig brain. Animal experiments were performed in accordance with the Guidelines for Animal Experiments at Semmelweis University.

### Cell Cultures

Brain capillary endothelial cells from 3 to 5 month-old Wistar rats were prepared and seeded on extracellular matrix coated glass coverslips as described earlier [23]. Cultures were kept in DMEM containing 17% plasma-derived bovine serum (First Link, UK), supplemented with 2 mM glutamine, 80 µg/ml heparin, 150 µg/ml endothelial cell growth supplement (Sigma), antibiotics, and trace factors (vitamin C, selenium, insulin, transferrin and glutathione). After reaching confluence, experiments were performed on 6–10 days old primary cultures.

Primary neuron-enriched cultures were prepared from E17 Wistar rat embryos, plated on 6 mm glass coverslips coated with poly-l-ornithine plus laminin in 12-well plates or on Petri dishes coated with poly-d-lysine, and maintained in Neurobasal medium (Invitrogen, Carlsbad, CA) with 2% B27 supplement (Invitrogen) and 2 mM glutamine for 8–12 days at 37 °C, 5% CO_2_ without feeding [24].

### Measurements of Mitochondrial Calcium Transients

In situ mitochondrial and cytoplasmic calcium measurements were performed in brain capillary endothelial cell cultures (at 7–8 days in vitro) by X-Rhod-1-AM (Molecular Probes, Eugene, OR, USA), localized mainly in mitochondria, a calcium sensitive dye with fluorescence microscopy as described earlier [25]. Briefly, mitochondrial calcium transients ([Ca^2+^]_m_) were analyzed using highpass spatial filtering of fluorescence images using the “the original X-rhod-1 mito filter.flt” in Image Analyst MKII (Image Analyst Software, Novato, CA, USA). Cytosolic calcium transients ([Ca^2+^]_c_) were analyzed on the same images left unfiltered by the measurement of X-Rhod-1 fluorescence of the nuclear region in the cell [25]. Half decay time (τ_1/2_) was defined as the time between peak of the transient and the intensity decaying to half of the peak amplitude.

Fluorimetric measurements were performed to examine potential optical or chemical interaction of vinpocetine and calcium sensitive fluorescent dyes (X-Rhod-1 and Fura-FF), to exclude the possibility of recording artefacts due to the change in fluorescence. Fluorescence of vinpocetine in aqueous solution was negligible both in the range of Fura-FF emission 510 nm (excitation: 340/380 nm) and in the range of X-Rhod-1 emission 570 nm (excitation: 535 nm). 30 μM vinpocetine had no effect on the fluorescence of hydrolyzed X-Rhod-1 or Fura-FF dyes either in high calcium (40 μM) containing or in calcium free intracellular solution (pH 7.4, 37 °C).

### Measurement of Delayed Ca^2+^ Deregulation in Isolated Primary Rat Brain Neurons

Time-lapse fluorescence microscopy of rat primary cortical cultures was carried out using the above fluorescence microscope setup using a UAPO 20 × dry 0.75 NA lens. Cultures were incubated with Fura-FF-AM (3 µM) for 15 min at 37 °C and subsequently rinsed with superfusion medium. Fura-FF fluorescence was ratio imaged using 340/10 (center/bandwidth in nm) and 380/10 exciters (Chroma, Rockingham, VT, USA), a 400DCLP dichroic mirror and a 470LP long pass emitter (Omega Optical, Brattleboro, VT, USA) [24].

### Isolation of Synaptosomes from Rat Brain

Synaptosomes were isolated from rat brain cortex as described earlier [26]. The synaptosomal fraction was obtained after sucrose (0.8 M) gradient centrifugation. Sucrose was diluted by ice cold distilled water to 0.32 M and synaptosomes were sedimented with 20,000×*g* for 20 min. The pellet’s protein concentration was adjusted to about 20 mg/ml, and synaptosomes were kept on ice until use.

Most of the experiments were performed in standard extracellular medium composed of (mM): 140 NaCl, 3 KCl, 2 MgCl_2_, 2 CaCl_2_, 10 PIPES pH 7.38 and 10 glucose. Incubations and measurements were done at 37 °C. Synaptosomes retained their basic bioenergetic parameters for at least 6 h.

### Isolation of Rodent Brain Mitochondria

Guinea pig mitochondria were isolated using discontinuous percoll gradient as described [27]. Mitochondria were incubated in the following incubation buffer (mM): 125 KCl, 20 HEPES, 2 K_2_HPO_4_, 1 MgCl_2_, 0.1 EGTA, at pH 7.0 adjusted by KOH. Incubation buffer was supplemented with 0.025 w/v % fatty acid free BSA. To use BSA was necessary, because mitochondria may lose their functional parameters very quickly as a consequence of liberated fatty acids [28].

### Oxygen Consumption of Synaptosomes and Mitochondria

Oxygen consumption of synaptosomes were measured by high resolution respirometry Oxygraph-2K (Oroboros Instruments, Innsbruck, Austria) [29] at 37 °C in 2-ml chambers. Data were digitally recorded and analyzed; oxygen flux was calculated as the negative temporal derivative of the oxygen concentration, *c*O_2_(*t*). Oxygen sensors were calibrated routinely in air saturated and oxygen depleted media. Synaptosomal protein concentration was 2 mg/ml, mitochondrial protein concentration was 0.1 mg/ml.

### Measurement of Mitochondrial Membrane Potential (Δψ_m_)

Mitochondrial membrane potential was measured by a custom-made tetraphenylphosphorium (TPP^+^) electrode [30]. TPP^+^ is a lipophilic membrane permeable cation. With the TPP^+^ electrode the extramitochondrial concentration of TPP^+^ was measured as described earlier. Knowing the total concentration of the TPP^+^ Δψ_m_ was calculated using the Nernst equation.

### Measurement of H_2_O_2_ Production in Mitochondria and in Synaptosomes

H_2_O_2_ released from mitochondria was detected with horseradish peroxidase (5U/2 ml) and Amplex Ultrared (3 μM) [31]. As a result of peroxidase action Amplex Ultrared was converted to fluorescent resorufin in the presence of mitochondria (0.1 mg/ml), and measured using PTI Deltascan fluorescence spectrophotometer (550 nm excitation, 585 nm emission wavelengths; Photon Technology International, Lawrenceville, NJ, USA). Each measurement was calibrated with known amounts of H_2_O_2_ at the end of the experiment.

### Measurement of Mitochondrial Ca^2+^ Uptake

Mitochondria (0.05 mg/ml) were incubated in the following reaction medium (mM): 8 KCl, 110 K-gluconate, 10 NaCl, 10 HEPES, 2 KH_2_PO_4_, 4 MgCl_2_, 10 mannitol, 5 glutamate, 5 malate, 3 ATP, 0.25 ADP pH 7.25 (KOH) supplemented with 0.025% BSA. The free Ca^2+^ concentration was calculated with the WinMAXC software ([32, 33]. Ca^2+^ (CaCl_2_) was given to mitochondria in the form of 12.5 μM Ca^2+^ pulses. The Ca^2+^ level of the medium was followed by Calcium-Green 5 N (K_D_ = 4.29 μM). Wavelengths for Calcium-green fluorescence were 505 nm excitation and 535 nm emission, respectively.

### Measurement of Mitochondrial Swelling

Swelling of isolated mitochondria can reflect permeability pore opening (see [33, 34]) and was followed by light scattering at 590 nm in parallel with mitochondrial Ca^2+^ uptake using double excitation, double emission mode of PTI Deltascan spectrofluorimeter. At the end of each measurement alamethicin (a pore forming peptide 80 μg/2 ml) was added to obtain maximal swelling.

### Kinetic measurement of Mitochondrial ATP Synthesis

Mitochondrial ATP formation was monitored by a combined enzymatic system comprising of hexokinase and glucose-6-phosphate dehydrogenase, as described earlier [35, 36]. Mitochondria (0.05 mg/ml) were incubated in the medium described in the isolation procedure. This medium was supplemented with 3 mM NADP^+^, 1.5 U hexokinase, 0.5 U glucose-6-phosphate dehydrogenase, 5 mM glucose, 2 mM ADP and 200 µM AP5 (P^1^,P^5^-Di(adenosine-5′) pentaphosphate), an inhibitor of adenylate kinase) in 2 ml total volume (Melnick et al. 1979). In the presence of mitochondria, ADP and respiratory substrates glutamate *plus* malate ATP was formed, which mostly left the mitochondria via the adenine nucleotide translocase. ATP phosphorylated glucose (to glucose-6-phosphate) by hexokinase. The resulted product glucose-6-phosphate was oxidized to 6-phosphogluconate by glucose-6-phospate dehydrogenase with the concomitant reduction of NADP^+^ to NADPH. Thus, NADPH formation was stoichiometrically equal to ATP released from the mitochondria. The absorbance of NADPH (ɛ = 6220 M^−1^ cm^−1^) was recorded at 340 nm, 37 °C using a JASCO V-650 spectrophotometer (ABL&E-JASCO, Tokyo, Japan). ATP standards were used for calibration.

### Measurement of ATPase Activity

ATP hydrolyzed by mitochondria in the absence of respiratory substrates was measured by a combined enzyme assay [37]. The standard assay medium was supplemented with NADH (300 μM), lactate dehydrogenase (2 U/ml), pyruvate kinase (PK; 2 U/ml), phosphoenolpyruvate (PEP; 2 mM), ATP (0.5 mM), and rotenone (1 μM). In the medium, ADP was phosphorylated to ATP in the presence of PK and PEP, then pyruvate was reduced to lactate with the concomitant oxidation of NADH to NAD^+^. Absorbance of NADH was monitored at 340 nm using a JASCO spectrophotometer. Measurements were calibrated with known amounts of ADP.

### Statistics

Statistical differences were evaluated with ANOVA in Sigmastat wherever multiple comparisons were made. Values are indicated as mean ± standard error, p < 0.05 were considered statistically significant.

## Results

### In Situ Mitochondrial Ca^2+^ Measurements in Brain Capillary Endothelial Cells

**Fig. 1:**
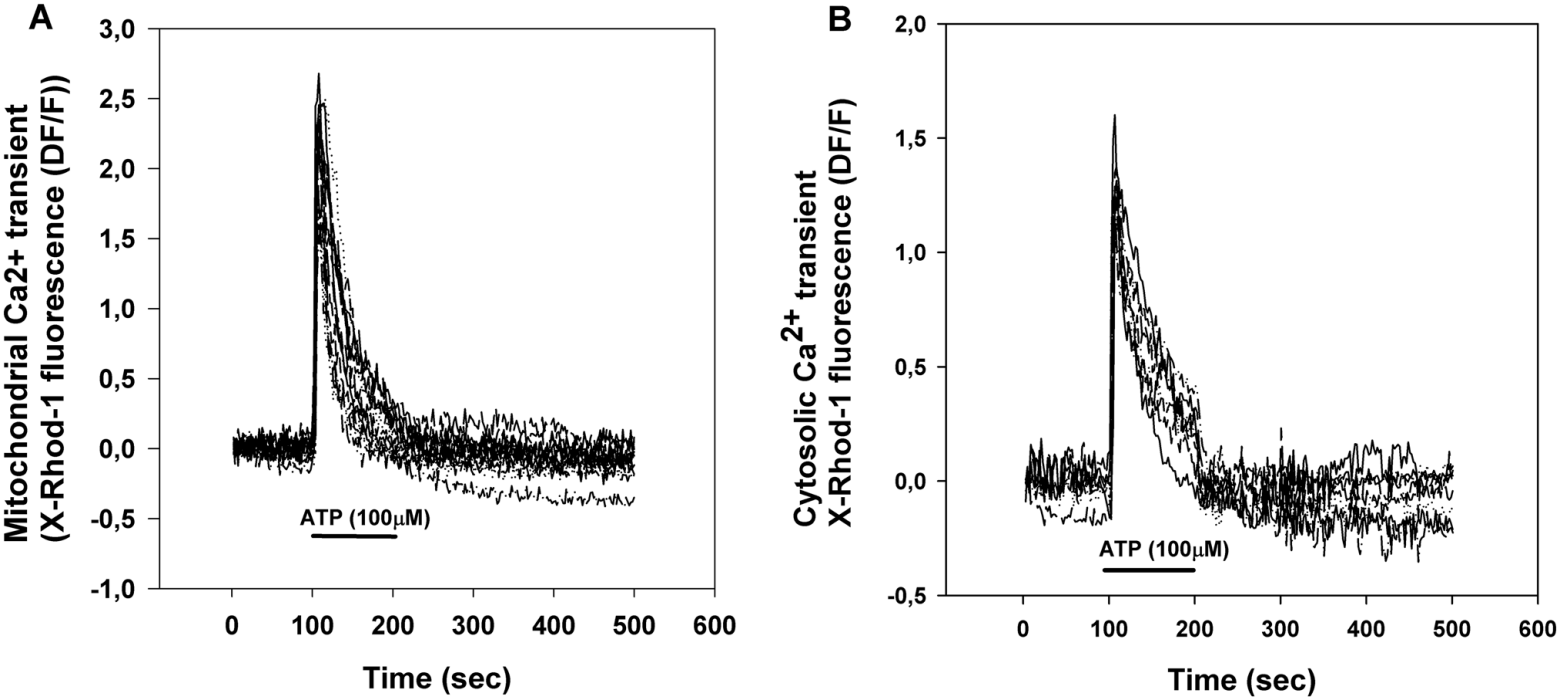
Mitochondrial (**a**) and cytosolic (**b**) calcium transients of primary brain capillary endothelial cells stimulated by the addition of 100 μM ATP. ATP was provided by superfusion at the indicated time. Mitochondrial and cytosolic Ca^2+^ transients were extracted from the same recordings using image processing. Pooled from 3 measurements, total cell number: 17

Brain capillary endothelial cells in primary culture were stimulated by the application of 100 μM ATP (Fig. 1). ATP triggers a transient rise of cytoplasmic [Ca^2+^] ([Ca^2+^]_c_, Fig. 1b) and mitochondrial [Ca^2+^] ([Ca^2+^]_m_, Fig. 1a) by activating purinergic receptors and IP3-mediated Ca^2+^ release from the endoplasmic reticulum [23]. Mitochondria take up Ca^2+^ from the cytoplasm by the calcium uniporter any Ca^2+^ is predominantly released from mitochondria by the Na^+^/Ca^2+^ exchanger.

In the stimulation paradigm depicted in Fig. 1, any change to Ca^2+^ ‘handling’ in mitochondria is indicated by the alteration of the peak amplitude or the half decay time of the mitochondrial Ca^2+^ transient. Alteration of the peak amplitude indicates changes both in the calcium influx and efflux while alteration of half decay time indicates change in the calcium efflux.

### Effect of Vinpocetine on ATP Induced [Ca^2+^]_m_ and [Ca^2+^]_c_ Transients

**Fig. 2:**
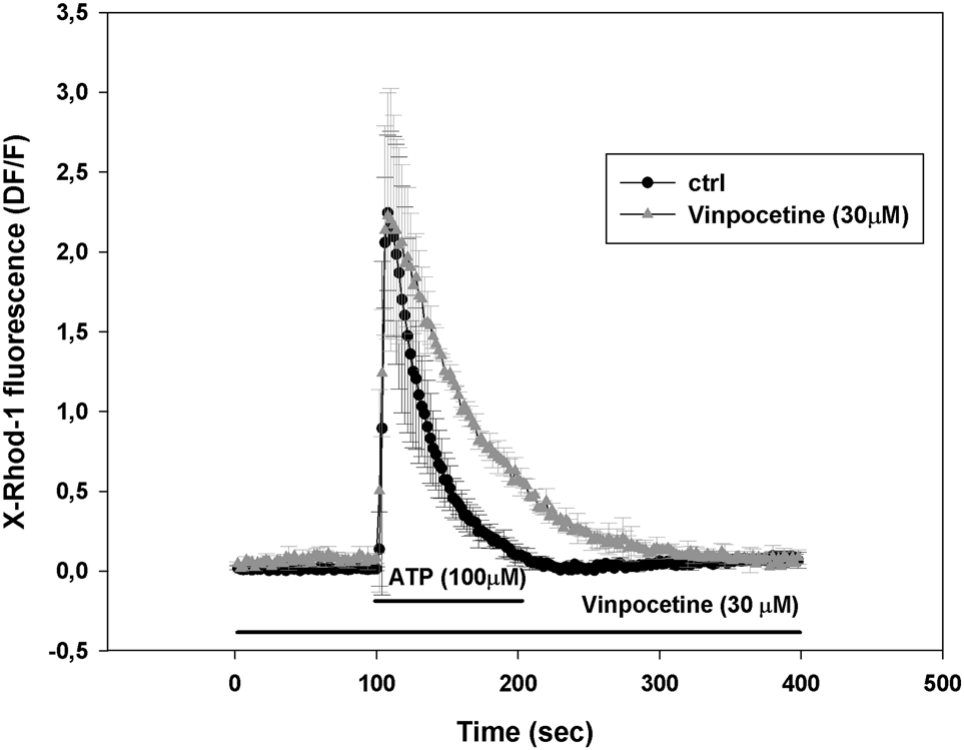
Effect of Vinpocetine (30 μM) pretreatment on the mitochondrial calcium transient. Data are expressed as mean ± SE of n = 3 experiments; ctrl, untreated control in matched culture preparations

Vinpocetine up to 10 μM had no significant effects on the parameters of [Ca^2+^]_m_, or [Ca^2+^]_c_ transients (not shown). However, 30 μM vinpocetine pretreatment significantly increased half decay time of the mitochondrial calcium transient (from τ_1/2_ = 23.5 ± 6.8 s to 42 ± 6.2 s, p < 0.05), but peak amplitude was not changed (from ampitude 2.24 ± 0.48 to 2.22 ± 0.77; given as relative fluorescence change from baseline, ΔF/F_0_) (Fig. 2).

Parameters of the cytosolic calcium transients were not significantly different (from τ_1/2_ = 33.3 ± 5.2 s to τ_1/2_ = 29 ± 8.69 s and from ampitude 1.33 ± 0.13 to 1.53 ± 0.15). Figure not shown.

### The Effect of Vinpocetine on Delayed Ca^2+^ Deregulation (DCD)

**Fig. 3:**
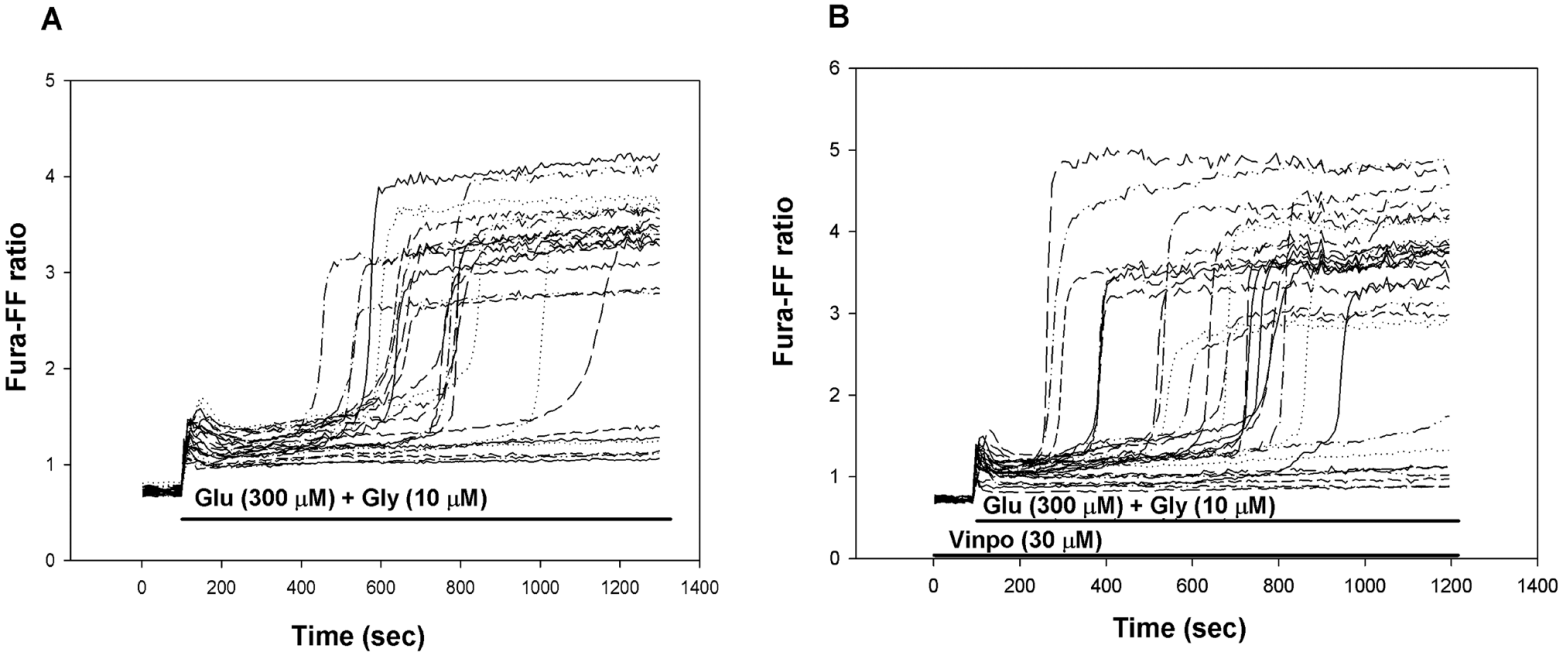
Delayed Ca^2+^ deregulation in primary cortical neurons in the absence (**a**) or presence (**b**) of vinpocetine (30 μM). Glutamate *plus* glycine (Glu + Gly) (**a**, **b**) and vinpocetine (vinpo) (**b**) were applied as indicated. The Fura-FF fluorescence excitation ratio reflects the cytosolic Ca^2+^ levels. Representative traces are shown

DCD is a model of glutamate toxicity, where excessive calcium load through Ca^2+^ permeable glutamate receptors could result in deterioration of calcium and energy homeostasis and eventually lead to cell death [38]. Primary cortical neurons were stimulated with glutamate (300 μM) *plus* glycine (10 μM) for 20 min in Mg^2+^-free solution and cytoplasmic [Ca^2+^] was measured using a low affinity calcium-sensitive fluorescent dye Fura-FF in single cells.The glutamate-induced initial Ca^2+^ peak was followed by a transient plateau where the cytosolic [Ca^2+^] remained lower than the peak, but higher than before the stimulation. The length of this period had large cell to cell variations and was followed by a sharp, irreversible elevation of [Ca^2+^]_c_ termed as DCD (Fig. 3a). Vinpocetine pretreatment (Fig. 3b) was unable to protect cells against DCD. There was neither a change in the mean time of DCD, nor in the fraction of cells exhibiting DCD within the timeframe of the experiment (20 min).

### The Effects of Vinpocetine on Synaptosomal Oxygen Consumption

**Fig. 4:**
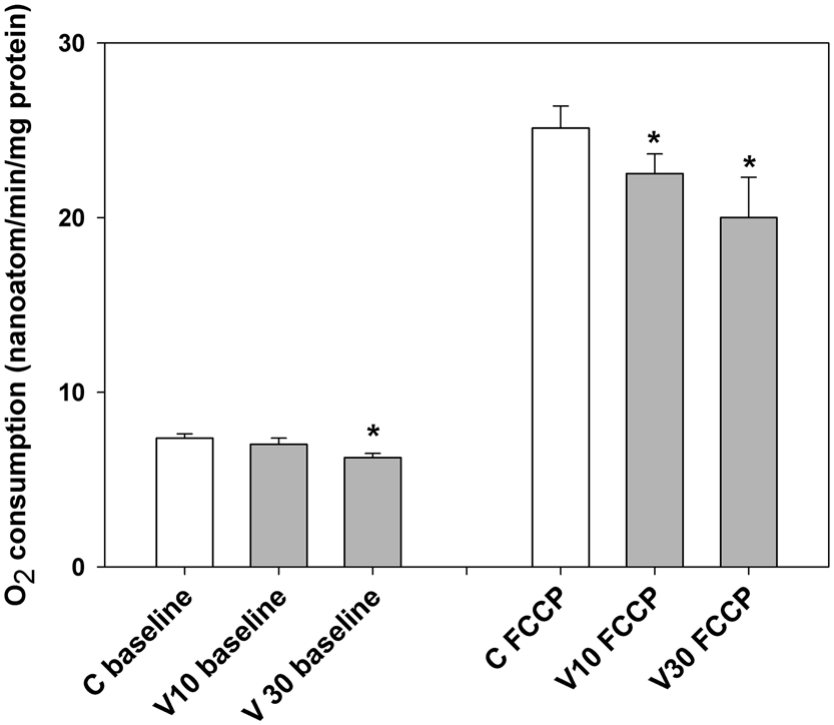
The effect of vinpocetine on the O_2_ consumption of synaptosomes. Synaptosomes were preincubated in glucose containing standard medium for 5 min under control conditions (C, white bars), or in the presence of vinpocetine (V, grey bars, 10 or 30 μM). First basal respiration (C baseline, V10 baseline, V30 baseline) was measured and then respiration was stimulated by the uncoupler FCCP (500 nM, C FCCP, V10 FCCP, V30 FCCP). * indicate significant difference (p < 0.05, n > 4) from the corresponding control measured in the absence of vinpocetine

Oxygen consumption of synaptosomes reflects substrate oxidation by in situ synaptosomal mitochondria, whereas contaminating, free mitochondria are assumed to be damaged and metabolically silent in the presence of mM extracellular Ca^2+^. Vinpocetine inhibited both resting and uncoupler-stimulated respiration of nerve endings. energized by glucose (Fig. 4). Replacing glucose by lactate resulted in similar results (data not shown).

### Effects of Vinpocetine on Synaptosomal H_2_O_2_ Production

**Fig. 5:**
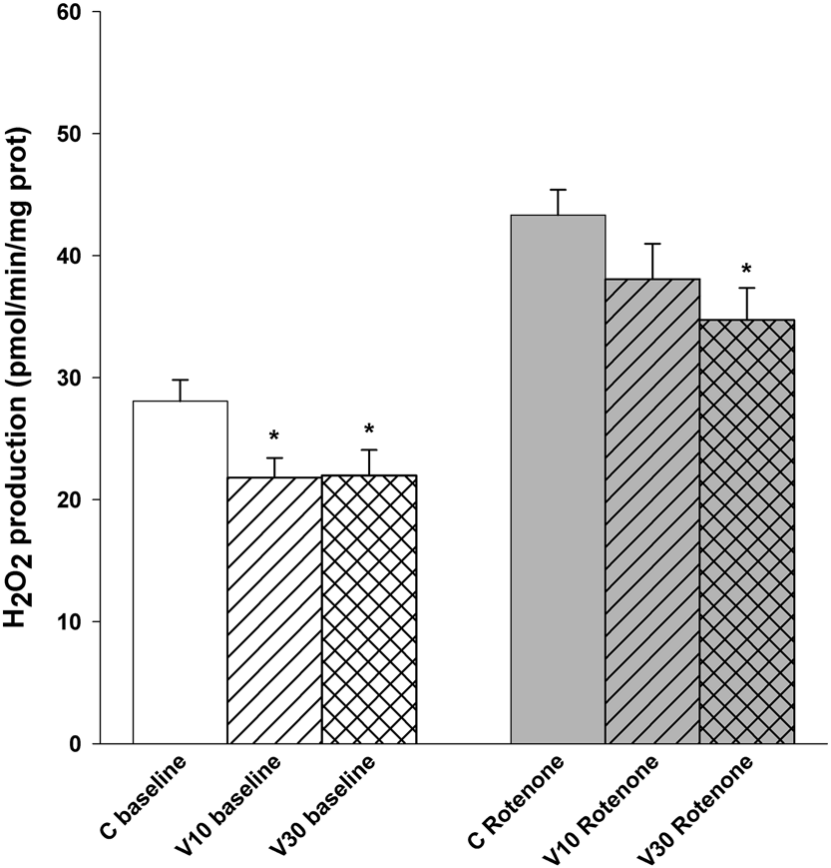
Effect of vinpocetine on H_2_O_2_ production of synaptosomes. Synaptosomes were preincubated in glucose-containing standard medium for 5 min under control conditions (C) or with vinpocetine (10 or 30 μM, hatched and cross hatched bars). Recording of baseline H_2_O_2_ formation was started after addition of Amplex Ultrared and horseradish peroxidase. After 200 s rotenone (1 μM, gray bars) was given. * indicate significant difference (p < 0.05, n > 4) compared to the corresponding control measured in the absence of vinpocetine

In synaptosomes, ROS production can primarily be attributed to mitochondria. To address the effects of vinpocetine on ROS generation, cortical synaptosomes supplied with glucose were preincubated with vinpocetine (10 or 30 μM) for 5 min. Vinpocetine lowered H_2_O_2_ release by 22%. In the presence of the complex I inhibitor rotenone-vinpocetine also significantly decreased H_2_O_2_ production by 19.7% (Fig. 5).

### Effects of Vinpocetine on Mitochondrial Membrane Potential

In isolated guinea pig brain mitochondria Δψ_m_ was measured with TPP^+^-electrode. Mitochondria were energized either by glutamate *plus* malate or succinate. After the development of Δψ_m_ vinpocetine (30 μM) was given. Vinpocetine decreased Δψ_m_ in glutamate *plus* malate supported mitochondria by 4.0 ± 0.6 mV. In succinate supported mitochondria similar depolarization was detected (data not shown).

### Effects of Vinpocetine on the Respiration of Isolated Guinea Pig Brain Mitochondria

**Fig. 6:**
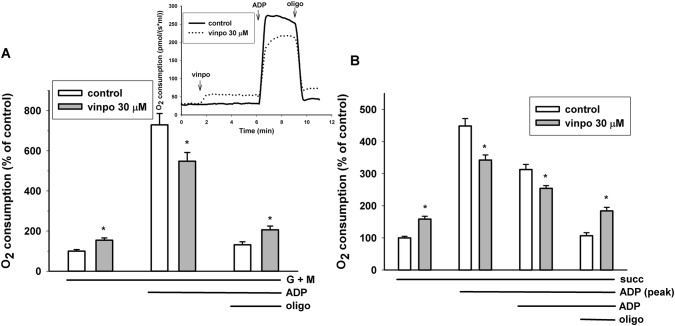
Effects of vinpocetine on mitochondrial respiration. Mitochondria supported by glutamate *plus* malate respiratory substrates (**a**) or by succinate (**b**) were incubated in standard mitochondrial medium. Vinpocetine (vinpo; 30 μM), ADP (2 mM) and oligomycin (oligo; 5 μM) were added as indicated in the original traces of the inset. The effects of vinpocetine (gray bars) were compared to the control (white bars, vehicle treated). Data are % of mean control oxygen consumption. Mean ± SE; n > 5; * significant difference from the corresponding solvent controls

Isolated mitochondria are intact cellular organelles with complex bioenergetic functions, e.g. membrane potential, ATP synthesis and Ca^2+^ transport. Brain mitochondria were supported with complex I (glutamate *plus* malate) or complex II (succinate) substrates. Adding vinpocetine during resting respiration (state 2, substrate only) stimulated oxygen consumption. In contrast in the presence of ADP (state 3 respiration) the rate of oxygen consumption was decreased by vinpocetine irrespective of the substrates applied (Fig. 6). Similarly, if vinpocetine was added after ADP, there was an immediate decrease in the respiration rate (not shown). Addition of oligomycin decreased the rate of respiration, but in the presence of vinpocetine the remaining respiration rate (leak respiration) was always higher than under control conditions, indicating the uncoupling effect of this vinca derivative.

### Effects of Vinpocetine on H_2_O_2_ Formation in Isolated Mitochondria

Usually there is a close correlation between the rate of oxidation and H_2_O_2_ production. The mitochondrial H_2_O_2_ formation has been investigated with glutamate *plus* malate and succinate substrates.

**Fig. 7:**
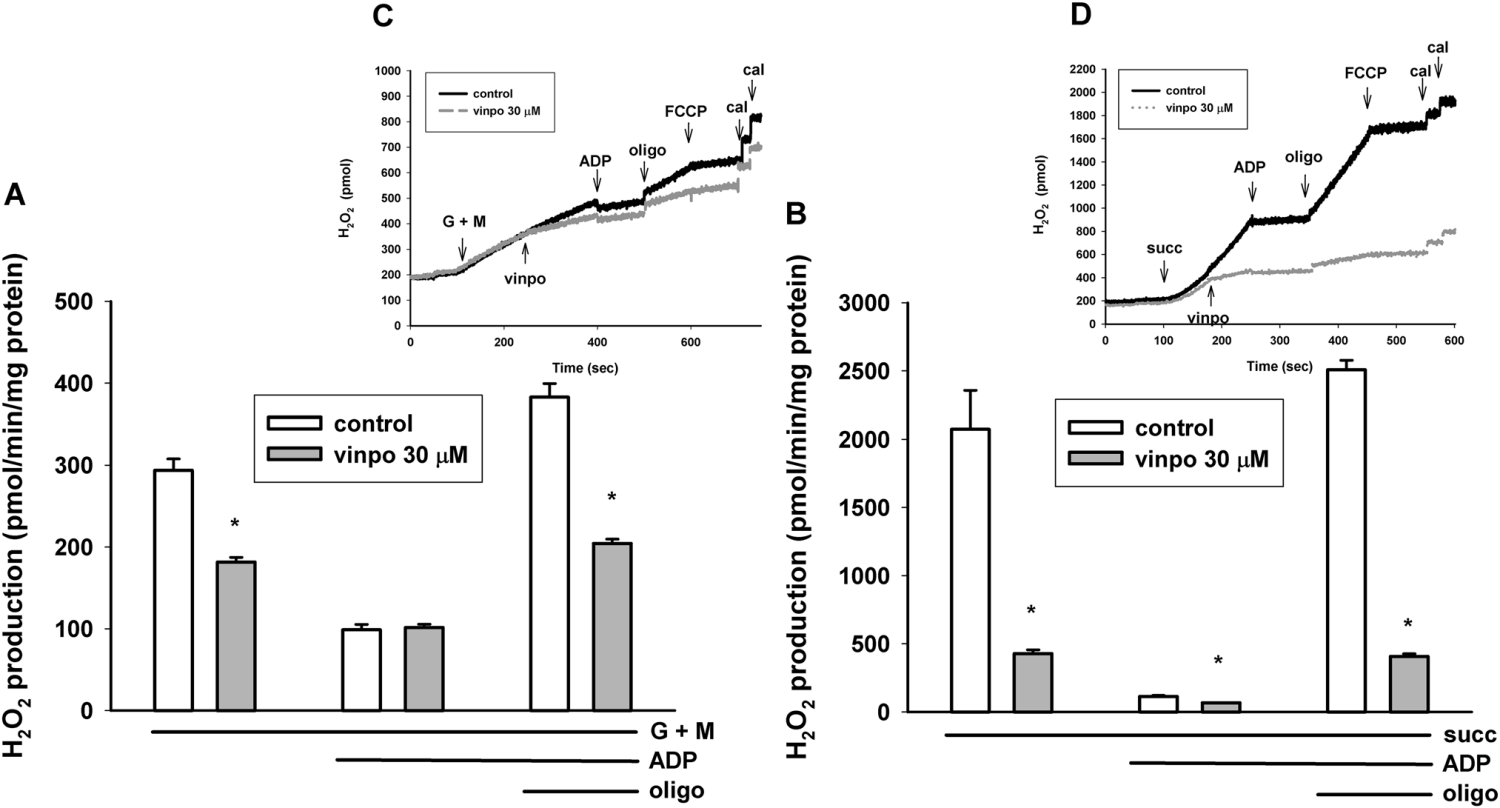
Effects of vinpocetine on mitochondrial H_2_O_2_ formation in glutamate *plus* malate (**a**) and succinate-supported (**b**) mitochondria. Mitochondria were incubated in the standard mitochondrial medium. Vinpocetine (vinpo; 30 μM), ADP (2 mM), oligomycin (oligo; 5 μM), and FCCP (250 nM) were given as indicated in the insets. At the end of each measurement 100 pmol H_2_O_2_ was added for calibration (cal). * indicates significant difference (p < 0.05, n > 4) from the corresponding vinpocetine-free controls

Using the complex I substrates glutamate *plus* malate, basal ROS production was only a fraction of that detected in succinate-supported mitochondria. This observation agrees with our and others previous studies [39, 40]. Vinpocetine also decreased the rate of H_2_O_2_ formation in the presence of glutamate *plus* malate (Fig. 7). Stimulation of respiration with ADP resulted in a decrease of ROS production irrespective of the presence or absence of vinpocetine. Inhibition of ATP synthesis with oligomycin stimulated H_2_O_2_ formation, and this increase was more modest (201% vs. 387%) in vinpocetine-treated mitochondria (Fig. 7a).

In succinate supported mitochondria ROS production was 2074 ± 282 pmol/min/mg protein. Under control conditions ADP dramatically decreased ROS production by 94.6% Vinpocetine added after succinate decreased H_2_O_2_ formation by 79.4%. Addition of the ATP synthesis inhibitor oligomycin stimulated H_2_O_2_ release by a factor of 22.2, while in the presence of vinpocetine only by a factor of only 6.1 (Fig. 3b).

### ATP Synthesis in Mitochondria

The most important bioenergetic function of mitochondria is ATP production. Alterations in state 3 respiration and membrane potential suggested a decreased capacity for ATP production. Measurement of ATP release from mitochondria in the presence of respiratory substrates and ADP provides information about this complex function.

**Fig. 8:**
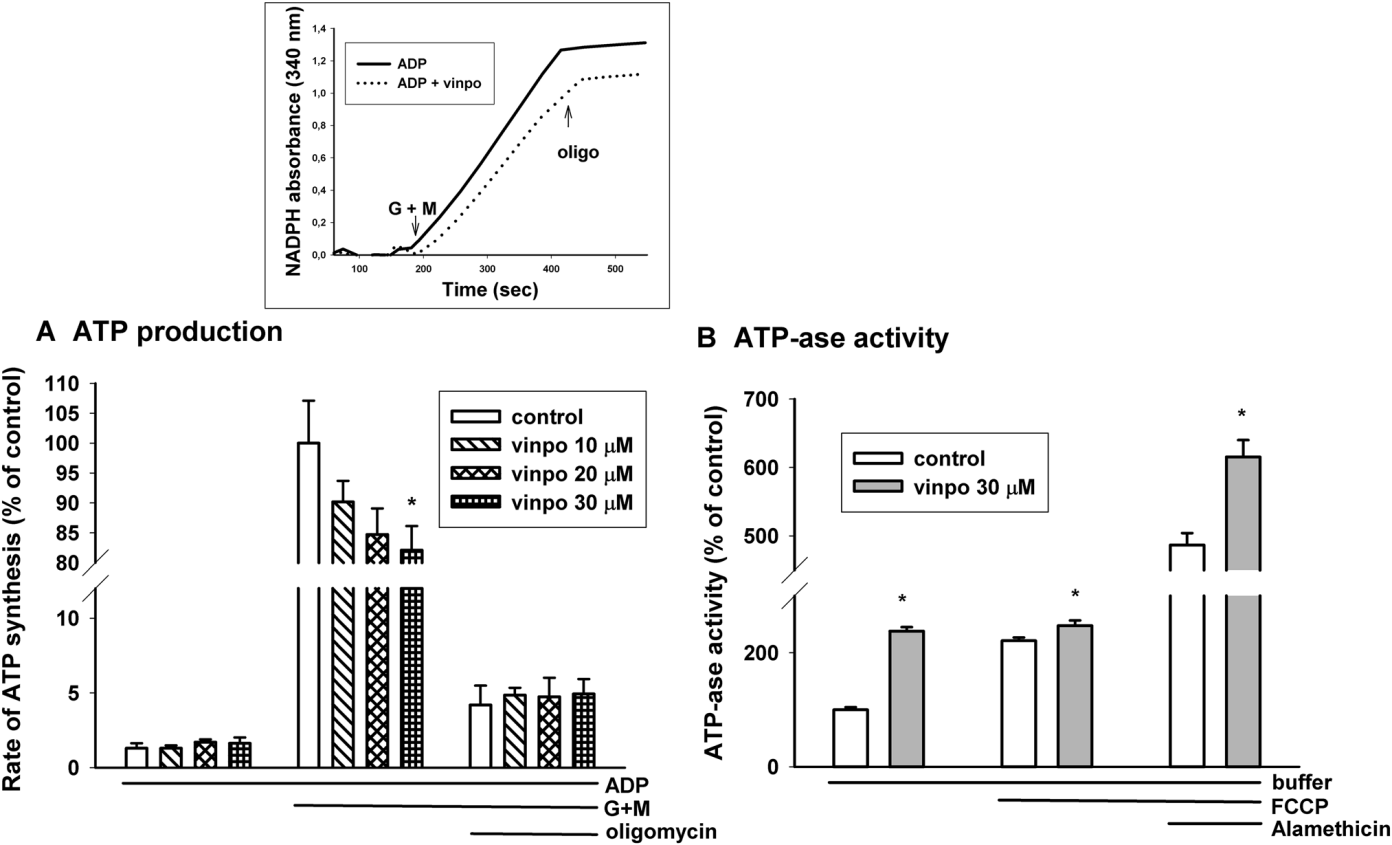
Effects of vinpocetine on mitochondrial ATP synthesis (**a**) and ATPase activity (**b**). The rate of ATP synthesis in glutamate *plus* malate (G + M) supported mitochondria (0.5 mg/ml mitochondrial protein was measured in standard medium supplemented with the inhibitor of adenylate kinase, ADP (2 mM) in the presence (hatched bars on (**a**) and dotted line on the inset) or absence (white bars on (**a**), solid line on the inset) of vinpocetine. At 200 s G + M initiated oxidative substrate-dependent ATP synthesis. Oligomycin (oligo; 5 μM) inhibited oxidative phosphorylation. ATPase activity (**b**) was measured on the basis of hydrolysis of exogenous ATP given to mitochondria. ATP hydrolysis was measured under resting conditions (buffer), in the presence of uncoupler FCCP (250 nM) and in the presence of alamethicin, a pore-forming antibiotics. Vinpocetine (vinpo; 30 μM; gray bars). * indicates significant difference (p < 0.05, n > 4) from the corresponding vinpocetine-free controls (white bars)

Mitochondrial ATP synthesis was measured in glutamate *plus* malate supported mitochondria. In the presence of glutamate *plus* malate vinpocetine evoked a dose-dependent inhibition of ATP synthesis (Fig. 8a). Applying 30 μM vinpocetine decreased ATP production by 17.9 ± 4.6%. Addition of oligomycin inhibited ATP production by about 95% indicating that most of the ATP production could be attributed to oxidative phosphorylation.

### ATPase Activity of Mitochondria

The formation of ATP by oxidative phosphorylation is influenced by many factors, e.g. transport of respiratory substrates, ADP, activity of respiratory complexes, activity of citric acid cycle enzymes. Therefore, for specific assessment of ATP synthase activity mitochondria were supported by ATP only and the rate of mitochondrial ADP release was measured. Mitochondria were incubated in the presence of vinpocetine (30 μM) or vehicle. In the presence of vinpocetine the rate of ADP hydrolysis more than doubled (increased by 137%). The uncoupler FCCP stimulated ADP hydrolysis and mitigated the difference between the effect of vinpocetine. In order to detect the maximal reverse activity of ATP synthase, mitochondria were permeabilized by the pore forming alamethicin. Vinpocetine also stimulated ATP hydrolysis in fully permeabilized mitochondria (Fig. 8b).

### Mitochondrial Ca^2+^ Uptake and Ca^2+^-Induced Ca^2+^ Release

**Fig. 9:**
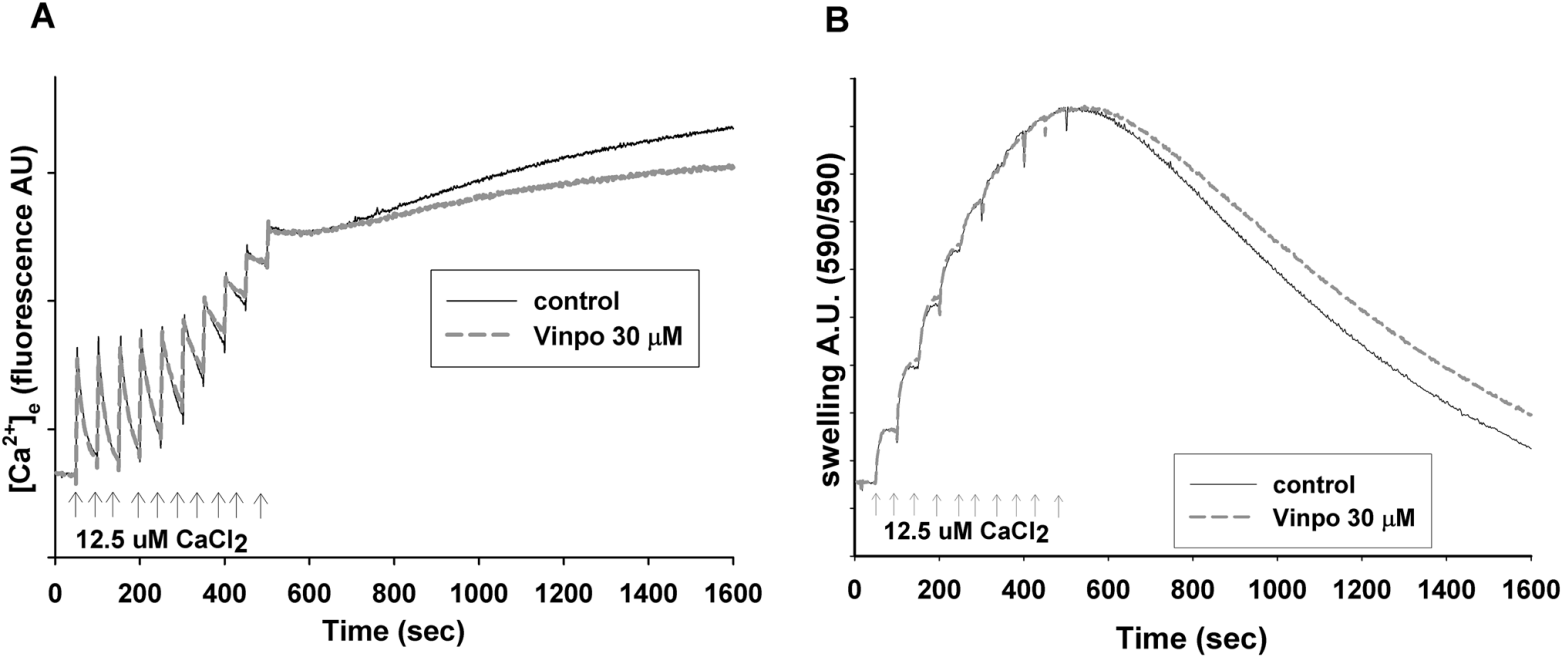
Effect of vinpocetine on mitochondrial Ca^2+^ handling. Effect of vinpocetine on the Ca^2+^ uptake capacity and mCICR. **a** Rat brain mitochondria (0.05 mg/ml) were incubated in reaction medium. CaCl_2_ pulses were added as indicated. The free [Ca^2+^] of the medium was detected by Calcium-Green 5 N fluorescence in the absence (control) or in the presence of vinpocetine (vinpo, 30 μM) as indicated. The loss of Ca^2+^ buffering capacity is followed by mCICR. Representative traces n > 3. Calcium induced swelling of mitochondria (**b**). Swelling of mitochondrial matrix was measured simultaneously with Ca^2+^ uptake by the light scattering method. More that swelling and the loss of Ca^2+^ buffering capacity were developed at the same time. Representative traces n > 3 from independent experiments

Another function of mitochondria is Ca^2+^-handling. To assess the Ca^2+^ uptake capacity, Ca^2+^ pulses (12.5 μM) were added and taken up by the mitochondria in 50 s intervals (Fig. 9a). The uptake was fast (V_max_ for the Ca^2+^ uniporter was 1200 nmol Ca^2+^/min/mg protein. After adding multiple Ca^2+^ pulses, the Ca^2+^ uptake capacity of mitochondria was gradually lost. When Ca^2+^ uptake stopped, mitochondria started to release the previously accumulated Ca^2+^ due to the mPTP opening (Ca^2+^-induced Ca^2+^ release (mCICR)). In the presence of vinpocetine (30 μM), the maximal Ca^2+^ uptake capacity of mitochondria remained unaltered, however the mCICR decreased.

### Effect of Vinpocetine on Ca^2+^-Induced Swelling of Mitochondria

In the above mCICR assay, in parallel with Ca^2+^ recording, swelling of mitochondria was measured from the same samples (Fig. 9b). In the presence of vinpocetine (30 μM) mitochondrial swelling (an indicator of mPTP opening) was decreased, indicated by the smaller decrease in light scatter after the onset of mCICR.

## Discussion

Vinpocetine was introduced to clinical therapy decades ago, and many possible molecules and structures were associated as targets for its beneficial effects. It is obvious that energy homeostasis is a crucial factor in the cell survival and can also determine the type of cell death. Although the binding of vinpocetine to the mitochondrial peripheral benzodiazepine receptor (PBR) has already been demonstrated [41, 42], systematic mitochondrial studies have not followed this early observation.

In the present study, the bioenergetic aspects of the effects of vinpocetine were investigated on primary brain capillary endothelial cells, neurons, isolated nerve terminals (synaptosomes) and in isolated mitochondria.

### Measurements in Cellular Systems

#### Ca^2+^ Transients in Primary Brain Capillary Endothelial Cells

Integrity of the blood brain barrier is an important factor in the maintenance of CNS homeostasis [43]. In brain capillary endothelial cells, purinergic signaling evoked cytoplasmic and mitochondrial Ca^2+^ transients (Fig. 1). In the presence of vinpocetine the peak height of the Ca^2+^ transient remained unaltered, however, the half decay time of the mitochondrial Ca^2+^ transient was increased, thus Ca^2+^ release was slower in the presence of vinpocetine (Fig. 2). The phenomenon may be attributed to inhibition of the mitochondrial Na^+^/Ca^2+^ exchange, the major route of the mitochondrial Ca^2+^ release [44]. This effect of vinpocetine is similar to that found with CGP-37157, a dedicated inhibitor of the mitochondrial Na^+^-dependent Ca^2+^ release [25]. CGP-37157 has neuroprotective effects [45]. Inhibition of ion channels is also in the spectrum of vinpocetine, e.g. it is an efficient blocker of the tetrodotoxin-sensitive voltage dependent Na^+^ channel. Nevertheless, there are further alternative explanations. Depolarization of mitochondria decreases the driving force of transport processes including the energy required for the forward operation of the Na^+^/Ca^2+^ exchanger [46].

### Delayed Ca^2+^ Deregulation (DCD) in Cortical Neurons

DCD is a model of glutamate toxicity, where excessive Ca^2+^ loads through Ca^2+^ permeable glutamate channels could result in deterioration of calcium and energy homeostasis. Vinpocetine did not affect DCD in primary cortical neurons. Our results somewhat differ from those published by others [12]. The reason of discrepancy is possibly attributed to the higher glutamate concentration applied in the present study (300 μM for 20 min vs. 25 μM for 30 min).

### Measurements on Isolated Nerve Terminals (Synaptosomes)

#### Oxygen Consumption Measurements

Oxygen consumption of synaptosomes is attributed to the substrate oxidation of in situ mitochondria [47]. Thus, glucose used as an energy supply for synaptosomes is metabolised in glycolysis and subsequently in the mitochondria. Compromised mitochondrial O_2_ consumption in synaptosomes energized by external glucose (Fig. 4) could be also explained by inhibition of either the glucose transport or glycolysis, besides mitochondrial effects. In order to rule out these possibilities, lactate was also used as respiratory substrate. Vinpocetine inhibited synaptosomal O_2_ consumption similarly in the presence of glucose or lactate, ruling out that this inhibition was be specific to the glucose metabolism. These observations directed our attention towards isolated mitochondria.

#### Release of H_2_O_2_ from Synaptosomes

Vinpocetine inhibited H_2_O_2_ formation in synaptosomes. Measurement of synaptosomal H_2_O_2_ production using Amplex Ultrared dye and horseradish peroxidase gives indirect information about the ROS homeostasis. The information are indirect, because (i) ROS sources other than mitochondria e.g. NADPH oxidases [47, 48], and nitrogen monoxide synthase (NOS) [49] can also be found in synaptosomes (ii) the cytoplasm possesses many enzymatic and nonenzymatic ROS scavenging systems [50, 51] and our detection system for H_2_O_2_ is extra-synaptosomal; in synaptosomes between the mitochondrial membranes and the plasma membrane of the synaptosomes both ROS generating and scavenging mechanisms can be found, therefore more direct information was acquired using isolated mitochondria.

### Measurements in Isolated Guinea Pig Brain Mitochondria

#### Effect of Vinpocetine on Mitochondrial Membrane Potential, Oxygen Consumption and H_2_O_2_ Formation

In isolated mitochondria membrane potential was slightly depolarized (4.0 ± 0.6 mV) by vinpocetine. This together with an increased respiration in the absence of ADP (Fig. 6) suggests an uncoupler-like effect that could be detected with both glutamate *plus* malate and succinate as respiratory substrates. In the absence of ADP (substrate only conditions) or in the presence of oligomycin the mitochondrial membrane potential is high and redox centers are very reduced. In this condition a high rate of H_2_O_2_ production was detected both with complex I and complex II substrates (Fig. 7). Even slight depolarization could exponentially decrease the ROS formation under these conditions [40, 52, 53]. Therefore, the ROS lowering effect of vinpocetine may be explained by mild uncoupling. The ADP stimulated respiration was strongly inhibited in the presence of vinpocetine. Like the depolarization in ADP-free conditions, the respiratory inhibition was also independent from what type of substrates (complex I or complex II) supported the mitochondria. This finding raises the possibility that either both complex I and II, or complex III, or complex IV or their combination were inhibited by vinpocetine.

#### ATP Synthetic and ATP Hydrolytic Rates in the Presence of Vinpocetine

Vinpocetine decreased the rate of ATP synthesis with glutamate *plus* malate as respiratory substrates, but increased the rate of ATP hydrolysis in the absence of respiration-supporting substrates. The concrete reasons behind a compromised mitochondrial ATP synthesis is generally rather obscure. In order to produce ATP efficiently, tens of mechanisms behind the four postulates of Mitchell should work perfectly and in synchrony. It is rather difficult to evaluate our findings from the aspect of neuroprotection. The slight decrease in the rate of ATP synthesis in the presence of vinpocetine might be attributed to the uncoupling detected in O_2_ consumption measurements. A less efficient ATP synthesis is not beneficial from the point of cell survival. On the other hand, an uncoupling-evoked decrease in membrane potential would decrease the rate of ROS production and could also accelerate ROS elimination by stimulating NADPH formation via the NADP^+^-dependent isocitrate dehydrogenase or glutamate dehydrogenase. Therefore, this phenomenon might improve the redox homeostasis in mitochondria and the cell. The increased ATP hydrolysis rate suggests that vinpocetine does not inhibit the reverse mode of ATP synthase (and perhaps does not inhibit its forward mode, either).

#### Seeking the Mechanism and Conclusions

In the present paper, we attempted to describe the effects of vinpocetine on selected mitochondrial functions in light of the well documented beneficial effects of the drug on brain functions. Various findings presented above are somewhat controversial. Some of them may first seem to be negative, e.g. inhibition of mitochondrial respiration and ATP synthesis. Some of the effects are subjects of various interpretations, e.g. mild uncoupling (its benefits are debated), the increased half time of Ca^2+^ release from endothelial cells, the possible inhibition of the mitochondrial Na^+^/Ca^2+^ exchanger, all may or may not have beneficial physiological effects. Finally, inhibition of the Ca^2+^-induced mitochondrial swelling, delayed CICR, and the very strong inhibition of the mitochondrial H_2_O_2_ release are all unequivocally positive.

Considering that vinpocetine does not display any severe side effects, we may conclude that the positive effects dominate under in vivo conditions. We also conclude that on the basis of the results presented above it is very likely that vinpocetine possesses more than one molecular target in mitochondria.
